# The Protection of Lactic Acid Bacteria Fermented-Mango Peel against Neuronal Damage Induced by Amyloid-Beta

**DOI:** 10.3390/molecules26123503

**Published:** 2021-06-08

**Authors:** Bao-Hong Lee, Wei-Hsuan Hsu, Chih-Yao Hou, Hao-Yuan Chien, She-Ching Wu

**Affiliations:** 1 Department of Horticulture, National Chiayi University, Chiayi 600355, Taiwan; s1072093@mail.ncyu.edu.tw; 2 Department of Food Safety/Hygiene and Risk Management, College of Medicine, National Cheng Kung University, Tainan 701401, Taiwan; whhsu@mail.ncku.edu.tw; 3 Center of Allergy and Mucosal Immunity Advancement, National Cheng Kung University, Tainan 701401, Taiwan; 4 Department of Seafood Science, National Kaohsiung University of Science and Technology, Kaohsiung 81157, Taiwan; chihyaohou@nkust.edu.tw; 5 Department of Food Science, National Chiayi University, Chiayi 600355, Taiwan

**Keywords:** mango peel, Neuron-2A cells, amyloid beta, *Lactobacillus acidophilus*, brain-derived neurotrophic factor

## Abstract

Mango peels are usually discarded as waste; however, they contain phytochemicals and could provide functional properties to food and promote human health. This study aimed to determine the optimal lactic acid bacteria for fermentation of mango peel and evaluate the effect of mango peel on neuronal protection in Neuron-2A cells against amyloid beta (Aβ) treatment (50 μM). Mango peel can be fermented by different lactic acid bacteria species. *Lactobacillus acidophilus* (BCRC14079)-fermented mango peel produced the highest concentration of lactic acid bacteria (exceeding 10^8^ CFU/mL). Mango peel and fermented mango peel extracts upregulated brain-derived neurotrophic factor (BDNF) expression for 1.74-fold in Neuron-2A cells. Furthermore, mango peel fermented products attenuated oxidative stress in Aβ-treated neural cells by 27%. Extracts of *L. acidophilus* (BCRC14079)-fermented mango peel treatment decreased Aβ accumulation and attenuated the increase of subG1 caused by Aβ induction in Neuron-2A cells. In conclusion, *L. acidophilus* (BCRC14079)-fermented mango peel acts as a novel neuronal protective product by inhibiting oxidative stress and increasing BDNF expression in neural cells.

## 1. Introduction 

Mango (*Mangifera indica* L.) is consumed as a dried fruit, as well as in juice, ice cream, and wine. Mango peel is usually discarded, resulting in environmental pollution caused by the carbohydrate (pectin, sucrose, and insoluble and soluble fiber), protein, and phytochemical (carotenoids and phenolic acid) content. Among these compounds, mangiferin is a functional antioxidant found in mango peel that promotes health. Mangiferin is a xanthone and is present in high levels in different parts (peel, leaves, and kernel). It is an antioxidant that has been demonstrated to protect cultured cortical neurons and organotypic slices against Aβ oligomers, attenuate oxidative stress and prevent mitochondrial dysfunction and neuronal injury [1]. Most importantly, mangiferin enhances hippocampal brain-derived neurotrophic factor (BDNF) levels and prevents cognitive deficits and hippocampal BDNF depletion related to neurotoxic agents [2,3].

Therefore, mango peel could be used to improve the functional properties of food. Mango peel powder has been used in biscuits, bread and several other types of foods [4]. Recently, mango has been used in lactic acid bacteria-fermented products [5,6], as well as fermentation with yeasts [7,8,9]. During gastrointestinal digestion and colonic fermentation, mango peel is able to increase polyphenolic compounds, leading to the transformation of mangiferin to norathyriol, mediated by microbiota [10,11]. Moreover, mango peel regulates gut microbiota, including promotion of Faecalibacterium, Roseburia, Eubacterium, Fusicatenibacter, Holdemanella, Catenibacterium, Phascolarctobacterium, Bifidobacterium, Collinsella, Prevotella and Bacteroides [12]. Various studies have demonstrated that the gut and central nervous system engage in crosstalk and that regulation of gut commensal flora could be a promising therapy for neurodegenerative diseases [13]. An increase in gastric vagus nerve activity by *Lactobacillus johnsonii* has been reported [14]. Moreover, the *Lactobacillus rhamnosus*-derived metabolites have potentials for ameliorating neuronal inflammation [15]. In this study, the optimal lactic acid bacteria for fermentation in mango peel was determined, and neuronal protection in Neuron-2A cells against amyloid beta (Aβ) induction was evaluated.

## 2. Results and Discussion

### 2.1. Mango Peel Fermentation by Lactic Acid Bacteria

**Figure 1:**
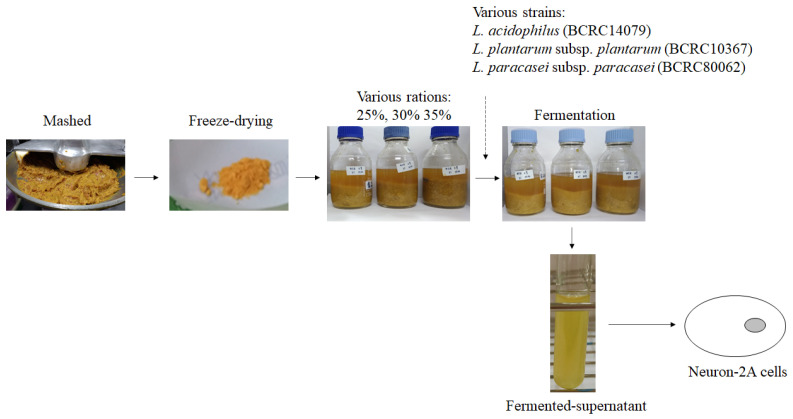
The fermented-process of mango peel by *L. acidophilus* (BCRC14079), *L. plantarum* subsp. *plantarum* (BCRC10367), and *L. paracasei* subsup. *paracasei* (BCRC80062).

*Lactobacillus acidophilus* (BCRC10695), *Lactiplantibacillus plantarum* subsp. *plantarum* NTU102, and *Lacticaseibacillus paracasei* subsp. *paracasei* NTU101 have been used in plant fermentation to increase antioxidative, anti-inflammatory, atherosclerosis-preventing, and antiobesity capabilities, to regulate lipid metabolism and to prevent acute gastric ulcers [16,17,18,19,20,21,22]. Moreover, some active ingredients have been found in lactic acid bacteria-fermented products, such as GABA and ACEI [23]. The mango peel fermentation process is shown in Figure 1. After mashing and freeze-drying, mango peel powder was dissolved to 25, 30, and 35% and fermented with different lactic acid bacteria species. After fermentation, the supernatant of fermented products was collected for treating Neuron-2A cells.

Titratable acid and pH value were determined in fermented-mango peel (30%) by different lactic acid bacteria species, including *L. acidophilus* (BCRC14079), *L. plantarum* subsp. *plantarum* (BCRC10367) and *L. paracasei* subsp. *paracasei* (BCRC80062).

**Figure 2:**
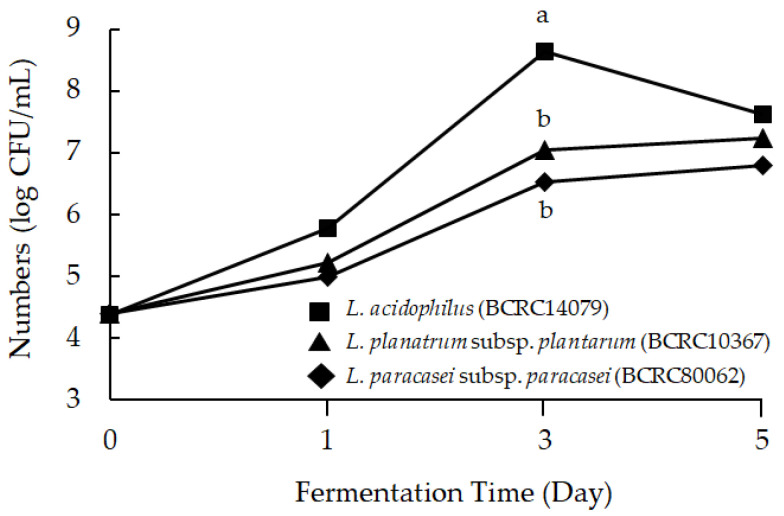
The change of bacterial number of lactic acid bacteria-fermented mango peel. Data are shown as means ± SD (*n* = 3). Significant differences are shown by various letters (*p* < 0.05).

**Figure 3:**
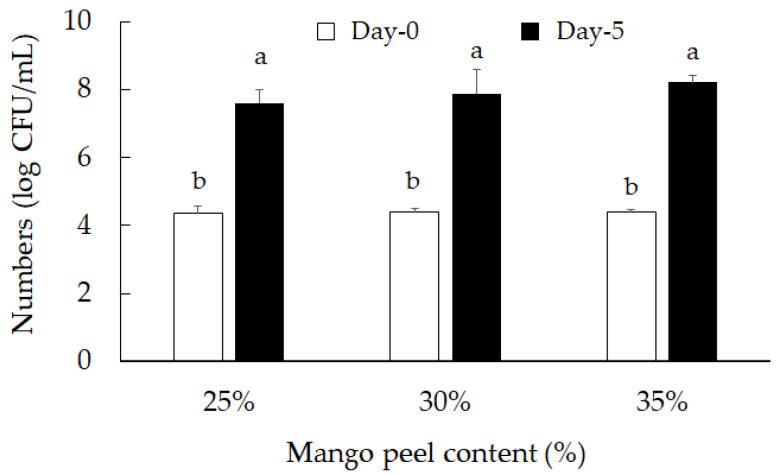
The change of bacterial number of *L. acidophilus* (BCRC14079)-fermented mango peel (various rations). Data are shown as means ± SD (*n* = 3). Significant differences are shown by various letters (*p* < 0.05).

pH decreased as fermentation time advanced (*p* < 0.05): the drop in pH and increase in titratable acid is due to the lactic acid bacteria and release of lactic acid. On the fifth day, the pH decreased from 6.55 ± 0.03 to 4.03 ± 006, 4.71 ± 005, and 4.33 ± 0.03 in *L. acidophilus*, *L. planatrum*, and *L. paracasei*, respectively (Table 1). The pH value in the *L. acidophilus*-fermented mango peel group dropped the fastest and decreased the most during the fermentation process. Figure 2 shows the experimental data of lactic acid bacteria species cell growth on medium with mango peel. The number of lactic acid bacteria increased after fermenting with mango peel for five days. *L. acidophilus* (BCRC14079) was the fastest growing strain, and reached the highest number of bacteria on the third day of fermentation. The number of viable lactic acid bacteria was significantly higher than the other two strains, exceeding 10^8^ CFU/mL. Differences in pH and titratable acid value were determined in *L. acidophilus* in solution with 25, 30, and 35% mango peel. After fermenting for five days, the pH value of *L. acidophilus* (BCRC14079)-fermented mango peel decreased and the titratable acid increased (Table 2). The 35% mango peel group had the lowest pH value (3.89 ± 0.02) and the highest titratable acid percentage (0.85 ± 0.09) during the fermentation process. Cell growth in *L. acidophilus* (BCRC14079)-fermented mango peel is shown in Figure 3. The results indicate that the number of viable lactic acid bacteria was similar in all three groups (25, 30, and 35% mango peel), which was near 10^8^ CFU/mL.

### 2.2. The Neuronal Protection of L. acidophilus (BCRC14079)-Fermented Mango Peel against Neuron-2A Cells Dysfunction Caused by Aβ Induction

**Figure 4:**
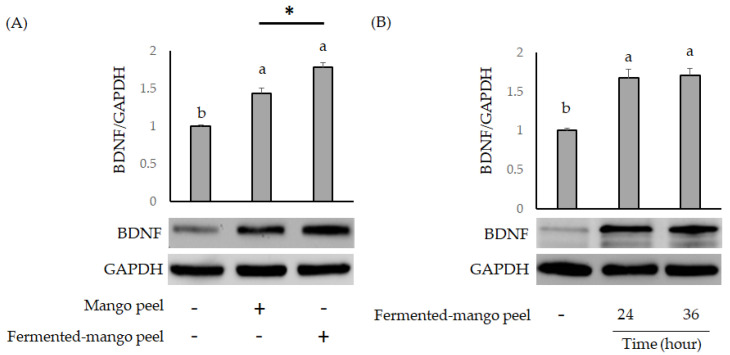
(**A**) The effects of mango peel and *L. acidophilus* (BCRC14079) fermented-mango peel extracts (100 μg/mL) on upregulation of BDNF in Neuron-2A cells after 36 h treatment. (**B**) The promotion of BDNF expression in Neuron-2A cells treated with *L. acidophilus* (BCRC14079) fermented-mango peel for 24 h and 36 h. Data are shown as means ± SD (*n* = 3). Significant differences are shown by various letters (*p* < 0.05). *p* values were also determined by Student’s *t*-test (*, *p* < 0.05).

The damaging effects of Aβ in Neuron-2A cells have been demonstrated in recent studies, including oxidative stress [24], mitochondrial function [25] and BDNF expression [26]. Both mango peel and fermented-mango peel extracts significantly promoted BDNF expression in Neuron-2A cells (Figure 4A), but this effect was not time-dependent (Figure 4B). BDNF expression was also analyzed by paired Student’s *t*-test, and there were significantly differences in the mango peel-treated group and fermented-mango peel extracts-treated group when using *t*-test to calculate the statistics.

**Figure 5:**
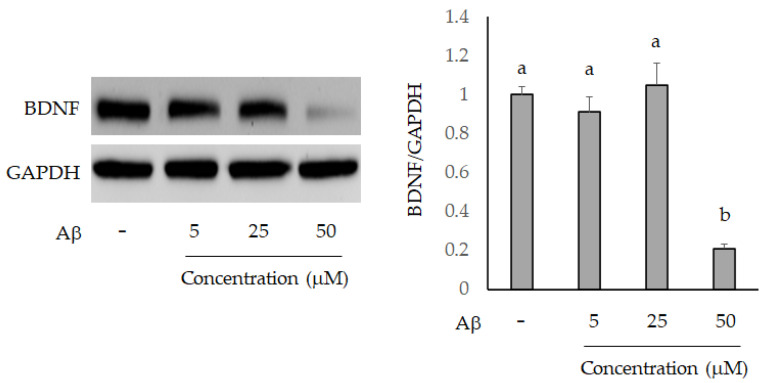
The impairment of BDNF in Neuron-2A cells treated by Aβ for various concentrations for 36 h. Data are shown as means ± SD (*n* = 3). Significant differences are shown by various letters (*p* < 0.05).

**Figure 6:**
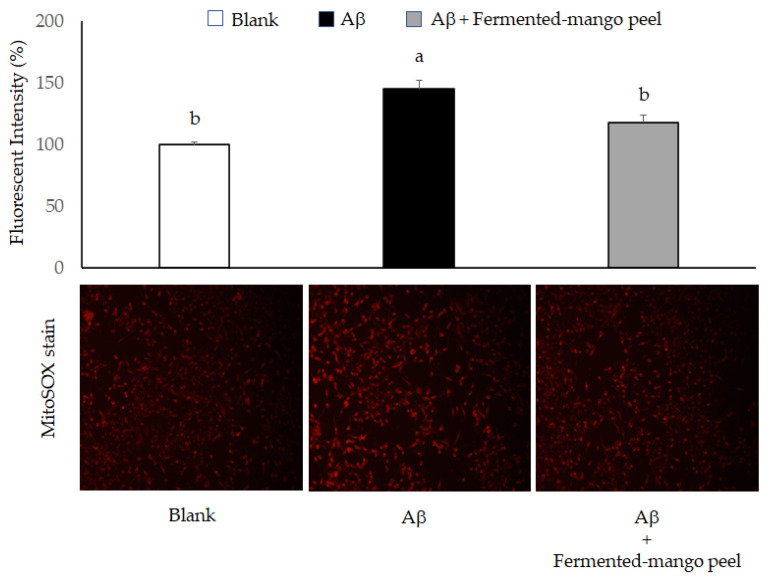
The attenuation of oxidative stress in Aβ-induced Neuron-2A cells by *L. acidophilus* (BCRC14079)-mango peel extracts (100 μg/mL) treatment for 36 h. Data are shown as means ± SD (*n* = 3). Significant differences are shown by various letters (*p* < 0.05).

**Figure 7:**
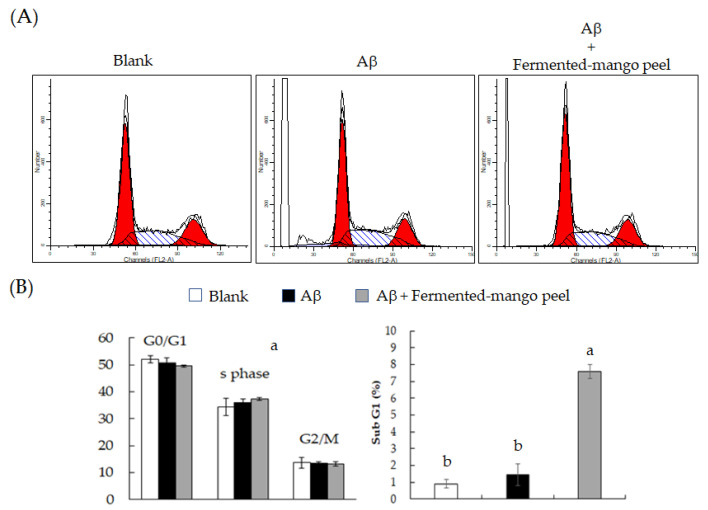
The levels of cell cycle and subG1 in Neuron-2A cells treated by Aβ with or without *L. acidophilus* (BCRC14079)-fermented mango peel extracts for 36 h. (**A**) Cell cycle distribution. (**B**) Quantitative results of cell cycle percentage distribution. Data are shown as means ± SD (*n* = 3). Significant differences are shown by various letters (*p* < 0.05).

The Neuron-2A cells were treated by 5, 25, and 50 μM Aβ for 36 h, and the BDNF level was evaluated using Western blot analysis. As shown in Figure 5, only 50 μM Aβ significantly inhibited BDNF expression compared to the control group. The Aβ peptide generates free radicals and leads to cell toxicity, such as protein and lipid oxidation, chromosomal damage and neuronal destruction [27]. Therefore, decreasing or scavenging the production of reactive oxygen species (ROS) is an important strategy to prevent Aβ-induced neurotoxicity in brain cells. Mitochondria are the main sources of ROS, and fluorogenic dye MitoSox could be an indicator for highly and selective detecting mitochondrial superoxide of live cells. MitoSox red stain revealed increased superoxide anion accumulation in Neuron-2A cells after treatment with Aβ for 36 h. However, this oxidative stress was attenuated by fermented-mango peel extract (100 μg/mL) treatment in Aβ-stimulated Neuron-2A cells (Figure 6). The inhibition of cell viability can result from the induction of apoptosis or cell growth suppression. Therefore, to clarify the cellular processes probably affected by *L. acidophilus* (BCRC14079)-fermented mango peel, the effect on the cell cycle was investigated using flow cytometry with propidium iodide (PI) staining. The results revealed that Aβ treatment did not affect the cell cycle at G0/G1, S, and G2/M phases, but weakly increased the subG1 level of Neuron-2A cells. Shanmuganathan et al. [24] and Sun et al. [26] reported that Aβ treatment (50 and 40 μM, respectively) induced cell death in Neuron-2A cells; however, significant toxic cellular effects were not found in Aβ treatment in Neuron-2A cells in this study (Figure 7A). In addition, treatment with *L. acidophilus* (BCRC14079)-fermented mango peel extracts (100 μg/mL) for 36 h suppressed an increase in subG1 caused by Aβ induction in Neuron-2A cells (Figure 7B).

**Figure 8:**
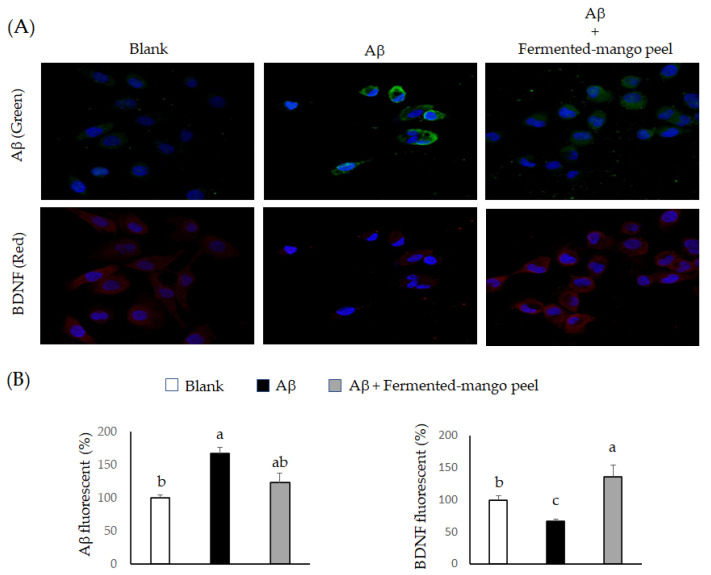
The regulations of *L. acidophilus* (BCRC14079)-fermented mango peel extracts on Aβ accumulation (green stain) and BDNF expression (red stain) in Neuron-2A cells treated with Aβ for 36 h. (**A**) ICC stain. (**B**) Quantitative results of fluorescence staining. Data are shown as means ± SD (*n* = 3). Significant differences are shown by various letters (*p* < 0.05).

The amyloid precursor protein (APP) in neuron cells is hydrolyzed by β- and γ-secretase to form Aβ [28]. Overexpression of APP led to Aβ accumulation and was related to AD occurrence [29,30]. Recently, *L. helveticus* NS8 has been found to improve behavioral, cognitive and biochemical aberrations [31], and *L. fermentum* NS9 has been indicated to restores physiological and psychological abnormalities [32]. Moreover, *Bifidobacterium longum* 1714 has also been evaluated for the potential of protecting against Alzheimer’s disease [33]. In this study, we further investigated the regulation of *L. acidophilus*-fermented mango peel extracts on BDNF expression in Aβ-induced Neuron-2A cells. Results are shown in Figure 8. *L. acidophilus* (BCRC14079)-fermented mango peel extracts significantly suppressed Aβ accumulation in vitro. A study found that *Lactobacillus* and *Bifidobacteria* both have ability to accelerate Aβ clearance through degrading enzymes [34]. We noticed a significant accumulation of Aβ and a downregulation of BDNF expression in Neuron-2A cells treated with Aβ for 36 h, as identified using ICC staining. This effect was attenuated by treatment with *L. acidophilus* (BCRC14079)-fermented mango peel extract, which helped the BDNF levels to recover (Figure 8). The mechanism of BDNF elevation was demonstrated in Neuron-2A cells treated with extracellular vesicles obtained from *L. plantarum* [35]. Collectively, *L. acidophilus*-fermented mango peel extracts alleviated Aβ deposition and protected Neuron-2A cells against toxic peptides. 

The effects of mango peel on microbiota shape in colon after anaerobic digestion were investigated. Results demonstrated that Faecalibacterium, Roseburia, Eubacterium, Fusicatenibacter, Holdemanella, Catenibacterium, Phascolarctobacterium, Buttiauxella, Bifidobacterium, Collinsella, Prevotella and Bacteroides genera were increased [12]. These bacteria have ability for generation of short-chain fatty acids in the colon, e.g., *Faecalibacterium prausnitzii* produces butyrate. Previous studies found that polyphenol of mango peel was bioconverted by gut microbiota, and mango peel could increase short-chain fatty acid level via predigestion in the human colon [36,37]. Moreover, butyrate is a neuron-protective agent against neuroinflammation in Alzheimer’s disease [38]. Taken together, lactic acid bacteria-fermented mango peel may be developed in functional products for improving neurodegenerative disorders in the future.

## 3. Materials and Methods

### 3.1. Chemicals

Fetal bovine serum (FBS) was purchased from Life Technologies (Auckland, New Zealand). Dimethyl sulfoxide (DMSO) was purchased from Wako Pure Chemical Industries (Saitama, Japan). Sodium dodecyl sulfate (SDS), Triton X-100, and trypsin were purchased from Sigma Chemical Co. (St Louis, MO, USA). Dulbecco’s modified Eagle’s medium (DMEM), penicillin and streptomycin were purchased from HyClone Laboratories (Logan, UT, USA). MitoSox^TM^-Red mitochondrial superoxide indicator was purchased from Thermo Fisher Scientific Inc. (Waltham, MA, USA). Lactobacilli MRS broth was purchased from Difco Laboratories (Detroit, MI, USA). 

### 3.2. Treatment and Fermentation of Mango Peel

Mango peel was made into a puree with a homogenizer and then freeze-dried to powder and stored at −80 °C. Next, the mango peel powder was formulated into different proportions (25, 30, and 35%) and fermented with lactic acid bacteria (*L. acidophilus* BCRC14079, *L. plantarum* subsp. *plantarum* BCRC10367, or *L. paracasei* subsp. *paracasei* BCRC80062) for five days. Lactic acid bacteria were subcultured with 1% inoculum in 10 mL sterilized MRS broth and subsequently inoculated into 400 mL mango peel solution in a triangle bottle. Counts of lactic acid bacteria, pH, and titratable acid were measured on the first, third, and fifth days. The three lactic acid bacteria strains were inoculated in MRS broth and grown under anaerobic conditions at 37 °C. The lactobacilli levels were measured by anaerobic cultivation on MRS plates. The fermented products were centrifuged to remove bacteria and mango peel residues. The supernatant was collected and filtered (0.22 μm), and the product was freeze-dried and stored at −80 °C until cellular and microbial experiments

### 3.3. Cell Culture

Cell culture and treatment Neuro-2A neuroblastoma cell line were obtained from the Bioresource Collection and Research Center (BCRC, Food Industry Research and Development Institute, Hsin Chu, Taiwan). Cells were grown in DMEM medium and supplemented with 10% heat-inactivated FBS, glutamine (2 mM), and L-glutamine (2 mM) at 37 °C in a humidified atmosphere of 95% air and 5% CO_2_. Cells were treated with Aβ (5, 25, and 50 μM) for 36 h or 48 h with or without *L. acidophilus* (BCRC14079)-fermented mango peel extracts (100 μg/mL) [39]. For Aβ induction, Aβ fragment 1–42 was obtained from Sigma Chemical Co. (CAT: A9810, St Louis, MO, USA) and preparation of Aβ oligomers was done according to [40]. Briefly, the lyophilized Aβ fragment was dissolved in dimethyl sulfoxide (DMSO) to 0.5 mM, and this solution was diluted to 50 μM using 20 mM HEPES buffer and incubated at 4 °C for 48 h, resulting in oligomer formation. The Aβ oligomers were observed by transmission electron microscopy (TEM) and used in cellular experiments. 

### 3.4. Western Blot

Cells were lysed in ice-cold lysis buffer containing 20 mM Tris-HCl (pH 7.4), 1% Triton-X-100, 0.1% SDS, 2 mM EDTA, 10 mM NaF, 1 mM phenyl-methanesulfonyl fluoride, 500 mM sodium vanadate and 10 mg/mL aprotinin overnight. The cell extract was centrifuged (12,000× *g*, 10 min) to obtain the supernatant. The supernatant was taken as the cell extract. The cell proteins were resolved on 10% SDS-PAGE and transferred to a polyvinylidene fluoride membrane. Membranes were blocked with 5% nonfat milk for 1 h and incubated with primary antibodies for 4 h. The membrane was then washed three times, each for 5 min, in PBS with Tween 20 (PBST), shaken in a solution of horseradish peroxidase-linked secondary antibody for 1 h, and washed three more times, each for 5 min, in PBST. Proteins were detected by enhanced chemiluminescent reagent (Millipore, Billerica, MA, USA).

### 3.5. MitoSOX-Red Stain

The assumption that mitochondria serve as the major intracellular source of ROS was based on experiments with isolated mitochondria, rather than on direct measurements in living cells. The MitoSOX™ Red mitochondrial superoxide indicator is a novel fluorogenic dye for the highly selective detection of superoxide in the mitochondria of viable cells. The MitoSOX™ Red reagent is a viable-cell permeant that rapidly and selectively targets the mitochondria. Once in the mitochondria, the reagent is oxidized by superoxide and exhibits red fluorescence. The reagent is readily oxidized by superoxide, but not by other ROS or reactive nitrogen species-generating systems, and the oxidation of the probe is prevented by superoxide dismutase. The oxidation product becomes highly fluorescent upon binding to nucleic acids. Mitochondrial superoxide is generated as a byproduct of oxidative phosphorylation. Briefly, cells were stained by MitoSOX™ Red reagent (5 μM) at 37 °C for 30 min, and the fluorescence intensity was measured using a confocal microscope (Leica Microsystems, Mannheim, Germany) [41].

### 3.6. Cell Cycle

Neuron-2A cells were PI stained for fluorescence-activated cell sorting (FACS) analysis. Cells (3 × 10^5^ cells/well) were seeded into sterile six-well plates. After 12 h incubation with Aβ, and with or without *L. acidophilus* (BCRC14079)-fermented mango peel extracts, the cells were detached using trypsin-EDTA, washed with PBS, collected through centrifugation (450 × *g*, 10 min), and stained with the PI staining solution containing 50 µg/mL PI, 0.5% (*w*/*v*) RNase A, and 0.1% (*v*/*v*) Triton-X-100. After incubation for 30 min at 4 °C in the dark, the cell cycle distribution was analyzed using flow cytometry on a FACS flow cytometer (Becton Dickinson & Co., Mountain View, CA, USA). A total of 100,000 events in each sample were acquired. The cell cycle distribution was determined using CellQuest Pro software (Becton Dickinson & Co., Franklin Lakes, NJ, USA) [42].

### 3.7. Immunocytochemistry Stain

Cells were stained with hoestest 33,342 for 30 min. After being rinsed twice with PBS, the cells were fixed with formaldehyde (3.7%) for 10 min, and the primary monoclonal antibody for 12 h at 4 °C. The sections were then washed with PBS and incubated with the secondary antibody (labeled with fluorescein isothiocyanate or rhodamine) in PBS for 1 h. The sections were then rinsed twice with PBS and cells were observed by confocal microscopy [43].

### 3.8. Statistical Analysis

Results were expressed as means ± SD. Comparisons among groups were made using one-way ANOVA. The differences between mean values in all groups were tested through Duncan’s multiple-range test (SPSS statistical software package, version 17.0, SPSS, Chicago, IL, USA). A *p*-value less than 0.05 was considered as a significant difference between means. BDNF levels were compared using two-tailed Student’s *t* test, and differences with a *p* value <0.05 were considered as statistically significant. 

## 4. Conclusions

Many chemicals have been used in neurodegeneration diseases, but they also exhibit side-effects. This study found a potential neuronal-protection effect of *L. acidophilus* (BCRC14079)-fermented mango peel mediated by inhibiting oxidative stress and promoting BDNF expression, with potential for development of pharmaceutical for neuronal protection.

## Figures and Tables

**Table 1:** The changes of pH and titratable acidity in fermented-mango peel (30%) by various lactic acid bacteria over the fermentation period.

**Fermentation Time (Day)**	***L. acidophilus* (BCRC14079)**	***L. planatrum* Subsp. *plantarum* (BCRC10367)**	***L. paracasei* Subsp.** ***paracasei* (BCRC80062)**
	**pH**		
0	6.55 ± 0.03 ^a^	6.55 ± 0.03 ^a^	6.55 ± 0.03 ^a^
1	5.13 ± 0.07 ^b^	5.74 ± 0.04 ^a,b^	5.48 ± 0.05 ^b^
3	4.45 ± 0.11 ^b^	5.06 ± 0.06 ^b^	4.89 ± 0.03 ^c^
5	4.03 ± 0.06 ^c^	4.71 ± 0.05 ^c^	4.33 ± 0.03 ^c^
**Fermentation Time (Day)**	**Titratable Acid (%)**		
0	0.07 ± 0.01 ^c^	0.07 ± 0.01 ^c^	0.07 ± 0.01 ^c^
1	0.13 ± 0.01 ^c^	0.09 ± 0.01 ^c^	0.11 ± 0.02 ^c^
3	0.43 ± 0.02 ^b^	0.23 ± 0.01 ^b^	0.27 ± 0.02 ^b^
5	0.56 ± 0.03 ^a^	0.37 ± 0.01 ^a^	0.42 ± 0.01 ^a^

Data are shown as means ± SD (*n* = 3). Significant differences are shown by various letters (*p* < 0.05).

**Table 2:** The changes of pH and titratable acid in *L. acidophilus* (BCRC14079)-fermented-mango peel products.

Mango Peel	pH	Titratable Acid (%)
25%	4.53 ± 0.05 ^a^	0.41 ± 0.02 ^c^
30%	4.11 ± 0.03 ^a,b^	0.68 ± 0.03 ^b^
35%	3.89 ± 0.02 ^b^	0.85 ± 0.09 ^a^

Data are shown as means ± SD (*n* = 3). Significant difference is shown by various letters (*p* < 0.05).

## Data Availability

Not applicable.

## Sample Availability

Samples of the compounds are available from the authors.

## Abbreviations

Aβ	amyloid beta
APP	amyloid precursor protein
BDNF	brain-derived neurotrophic factor
CNS	central nervous system
DMEM	Dulbecco’s modified Eagle’s medium
DMSO	dimethyl sulfoxide
FBS	fetal bovine serum
ROS	reactive oxygen species
SDS	sodium dodecyl sulfate

## References

[1] Feng S.T., Wang Z.Z., Yuan Y.H., Sun H.M., Chen N.H., Zhang Y. (2019). Mangiferin: A multipotent natural product preventing neurodegeneration in Alzheimer’s and Parkinson’s disease models. Pharmacol. Res..

[2] Kasbe P., Jangra A., Lahkar M. (2015). Mangiferin ameliorates aluminium chloride-induced cognitive dysfunction via alleviation of hippocampal oxido-nitrosative stress, proinflammatory cytokines and acetylcholinesterase level. J. Trace Elem. Med. Biol..

[3] Luo G.Q., Liu L., Gao Q.W., Wu X.N., Xiang W., Deng W.T. (2017). Mangiferin prevents corticosterone-induced behavioural deficits via alleviation of oxido-nitrosative stress and down-regulation of indoleamine 2,3-dioxygenase (IDO) activity. Neurol. Res..

[4] Chen Y., Zhao L., He T., Ou Z., Hu Z., Wang K. (2019). Effects of mango peel powder on starch digestion and quality characteristics of bread. Int. J. Biol. Macromol..

[5] Reddy L., Min J.H., Wee Y.J. (2015). Production of probiotic mango juice by fermentation of lactic acid bacteria. Korean J. Microbiol. Biotechnol..

[6] Liao X.Y., Guo L.Q., Ye Z.W., Qiu L.Y., Gu F.W., Lin J.F. (2016). Use of autochthonous lactic acid bacteria starters to ferment mango juice for promoting its probiotic roles. Prep. Biochem. Biotechnol..

[7] Kamassah A.K.Q., Saalia F.K., Fosu P.O., Mensah-Brown H., Sinayobye E., Tano-Debrah K. (2013). Fermentation capacity of yeasts using mango (*Mangiferia indica* Linn.) as substrate. Food Sci Qual. Manag..

[8] Reddy L.V., Reddy O.V.S. (2007). Production of ethanol from mango (*Mangifera indica* L.) fruit juice fermentation. Res. J. Microbiol..

[9] Li X., Chan L.J., Yu B., Curran P., Liu S.Q. (2012). Fermentation of three varieties of mango juices with a mixture of *Saccharomyces cerevisiae* and *Williopsis saturnus* var. mrakii. Int. J. Food Microbiol..

[10] Ordonez-Diaz J.L., Moreno-Ortega A., Roldan-Guerra F.J., Ortiz-Somovilla V., Moreno-Rojas J.M., Pereira-Caro G. (2020). In vitro gastrointestinal digestion and colonic catabolism of mango (*Mangifera indica* L.) pulp polyphenols. Foods.

[11] Hernandez-Maldonado L.M., Blancas-Benitez F.J., Zamora-Gasga V.M., Cardenas-Castro A.P., Tovar J., Sayago-Ayerdi S.G. (2019). In vitro gastrointestinal digestion and colonic fermentation of high dietary fiber and antioxidant-rich mango (*Mangifera indica* L.) “Ataulfo”-based fruit bars. Nutrients.

[12] Gutierrez-Sarmiento W., Sayago-Ayerdi S.G., Goni I., Gutierrez-Miceli F.A., Abud-Archila M., Rejon-Orantes J.C., Rincon-Rosales R., Pena-Ocana B.A., Ruiz-Valdiviezo V.M. (2020). Changes in intestinal microbiota and predicted metabolic pathways during colonic fermentation of mango (*Mangifera indica* L.)-based bar indigestible fraction. Nutrients.

[13] Bostanciklioglu M. (2019). The role of gut microbiota in pathogenesis of Alzheimer’s disease. J. Appl. Microbiol..

[14] Tanida M., Yamano T., Maeda K., Okumura N., Fukushima Y., Nagai K. (2005). Effects of intraduodenal injection of *Lactobacillus johnsonii* La1 on renal sympathetic nerve activity and blood pressure in urethane-anesthetized rats. Neurosci. Lett..

[15] Bravo J.A., Forsythe P., Chew M.V., Escaravage E., Savignac H.M., Dinan T.G., Bienenstock J., Cryan J.F. (2011). Ingestion of *Lactobacillus* strain regulates emotional behavior and central GABA receptor expression in a mouse via the vagus nerve. Proc. Natl. Acad. Sci. USA.

[16] Wu S.C., Su Y.S., Cheng H.Y. (2011). Antioxidant properties of *Lactobacillus*-fermented and non-fermented *Graptopetalum paraguayense* E. Walther at different stages of maturity. Food Chem..

[17] Liu C.F., Tseng K.C., Chiang S.S., Lee B.H., Hsu W.H., Pan T.M. (2011). Immunomodulatory and antioxidant potential of *Lactobacillus* exopolysaccharides. J. Sci. Food Agric..

[18] Liu C.F., Hu C.L., Chiang S.S., Tseng K.C., Yu R.C., Pan T.M. (2009). Beneficial preventive effects of gastric mucosal lesion for soy-skim milk fermented by lactic acid bacteria. J. Agric. Food Chem..

[19] Chiu C.H., Lu T.Y., Tseng Y.Y., Pan T.M. (2006). The effects of *Lactobacillus*-fermented milk on lipid metabolism in hamsters fed on high-cholesterol diet. Appl. Microbiol. Biotechnol..

[20] Pan T.M., Chiu C.H., Guu Y.K. (2002). Characterization of *Lactobacillus* isolates from pickled vegetables for use as dietary or pickle adjuncts. Foods Food Ingred. J. Jpn..

[21] Tsai Y.Y., Chu L.H., Lee C.L., Pan T.M. (2009). Atherosclerosis-preventing activity of lactic acid bacteria-fermented milk-soymilk supplemented with *Momordica charatia*. J. Agric. Food Chem..

[22] Lee B.H., Lo Y.H., Pan T.M. (2013). Anti-obesity activity of *Lactobacillus* fermented soy milk products. J. Funct. Foods.

[23] Tung Y.T., Lee B.H., Liu C.F., Pan T.M. (2011). Optimization of culture condition for ACEI and GABA production by lactic acid bacteria. J. Food Sci..

[24] Shanmuganathan B., Sathya S., Balasubramaniam B., Balamurugan K., Devi K.P. (2019). Amyloid-β induced neuropathological actions are suppressed by *Padina gymnospora* (Phaeophyceae) and its active constituent α-bisabolol in Neuro2a cells and transgenic *Caenorhabditis elegans* Alzheimer’s model. Nitric Oxide.

[25] Manczak M., Mao P., Calkins M., Cornea A., Arubala R.P., Murphy M.P., Szeto H.H., Park B., Reddy P.H. (2010). Mitochondria-targeted antioxidants protect against Abeta toxicity in Alzheimer’s disease neurons. J. Alzheimers Dis..

[26] Sun J., Yuan B., Wu Y., Gong Y., Guo W., Fu S., Luan Y., Wang W. (2020). Sodium butyrate protects N2a cells against Aβ toxicity in vitro. Media. Inflamm..

[27] Liu K., Liu P.C., Liu R., Wu X. (2015). Dual AO/EB staining to detect apoptosis in osteosarcoma cells compared with flow cytometry. Med. Sci. Mon. Basic Res..

[28] Wang X., Zhou X., Li G., Zhang Y., Wu Y., Song W. (2017). Modifications and trafficking of APP in the pathogenesis of Alzheimer’s disease. Fr. Mol. Neurosci..

[29] Seki T., Kanagawa M., Kobayashi K., Kowa H., Yahata N., Maruyama K., Iwata N., Inoue H., Toda T. (2020). Galectin 3-binding protein suppresses amyloid-β production by modulating β-cleavage of amyloid precursor protein. J. Biol. Chem..

[30] Esquerda-Canals G., Montoliu-Gaya L., Guell-Bosch J., Villegas S. (2017). Mouse models of Alzheimer’s disease. J. Alzheimer Dis..

[31] Liang S., Wang T., Hu X., Luo J., Li W., Wu X., Duan Y., Fin F. (2015). Administration of *Lactobacillus helveticus* NS8 improves behavioral, cognitive, and biochemical aberrations caused by chronic restraint stress. Neuroscience.

[32] Wang T., Hu X., Liang S., Li W., Wu X., Wang L., Jin F. (2015). *Lactobacillus fermentum* NS9 restores the antibiotic induced physiological and psychological abnormalities in rats. Benef. Microbes.

[33] Lee H.J., Lee K.E., Kim J.K., Kim D.H. (2019). Suppression of gut dysbiosis by *Bifidobacterium longum* alleviates cognitive decline in 5XFAD transgenic and aged mice. Sci. Rep..

[34] Azm S.A.N., Djazayery A., Safa M., Azami K., Ahmadvand B., Sabbaghziarani F., Sharifzadeh M., Vafa M.R. (2018). Lactobacillus and Bifidobacterium ameliorate memory and learning deficits and oxidative stress in Aβ (1-42) injected rats. Appl. Physiol. Nutr. Metab..

[35] Choi J., Kim Y.K., Han P.L. (2019). Extracellular vesicles derived from *Lactobacillus plantarum* increase BDNF expression in cultured hippocampal neurons and produce antidepressant-like effects in mice. Exp. Neurobiol..

[36] Sayago-Ayerdi S.G., Zamora-Gasga V.M., Venema K. (2019). Prebiotic effect of predigested mango peel on gut microbiota assessed in a dynamic in vitro model of the human colon (TIM-2). Food Res. Int..

[37] Sayago-Ayerdi S.G., Venema K., Tabernero M., Sarria B., Bravo L.L., Mateos R. (2021). Bioconversion by gut microbiota of predigested mango (*Mangifera indica* L)’Ataulfo’ peel polyphenols assessed in a dynamic (TIM-2) in vitro model of the human colon. Food Res. Int..

[38] Sun J., Xu J., Yang B., Chen K., Kong Y., Fang N., Gong T., Wang F., Ling Z., Liu J. (2020). Effect of *Clostridium butyricum* against mnicroglia-mediated neuroinflammation in Alzheimer’s disease via regulating gut microbiota and metabolites butyrate. Mol. Nutr. Food Res..

[39] Lee C.C., Lee B.H., Wu S.C. (2014). *Actinidia callosa* peel (kiwi fruit) ethanol extracts protected neural cells apoptosis induced by methylglyoxal through Nrf2 activation. Pharm. Biol..

[40] Domert J., Rao S.B., Agholme L., Brorsson A.C., Marcusson J., Hallbeck M., Nath S. (2014). Spreading of amyloid-β peptides via neuritic cell-to cell transfer is dependent on insufficient cellular clearance. Neurobiol. Dis..

[41] Hsu W.H., Lin Y.C., Chen B.R., Wu S.C., Lee B.H. (2018). The neuronal protection of a zinc-binding protein isolated from oyster. Food Chem. Toxicol..

[42] Han Y., Cui Z., Li Y.H., Hsu W.H., Lee B.H. (2016). In vitro and in vivo anticancer activity of pardaxin against proliferation and growth of oral squamous cell carcinoma. Mar. Drugs.

[43] Hsu W.H., Lee B.H., Pan T.M. (2015). Leptin-induced mitochondrial fusion mediates hepatic lipid accumulation. Int. J. Obesity.

